# Human in vitro airway models to assess point-of-contact respiratory toxicity of surfactants: development and evaluation of a repeated-exposure study design

**DOI:** 10.1093/toxsci/kfag146

**Published:** 2026-10-30

**Authors:** Monita Sharma, Andreas O Stucki, Sandra Verstraelen, Karen Hollanders, An Jacobs, Nuria Roldan, Sylvie Remy, Adam Bettmann, Amy J Clippinger

**Affiliations:** PETA Science Consortium International e. V, 70499 Stuttgart, Germany; PETA Science Consortium International e. V, 70499 Stuttgart, Germany; Environmental Intelligence Unit, Flemish Institute for Technological Research (VITO), Mol 2400, Belgium; Environmental Intelligence Unit, Flemish Institute for Technological Research (VITO), Mol 2400, Belgium; Environmental Intelligence Unit, Flemish Institute for Technological Research (VITO), Mol 2400, Belgium; PETA Science Consortium International e. V, 70499 Stuttgart, Germany; Environmental Intelligence Unit, Flemish Institute for Technological Research (VITO), Mol 2400, Belgium; PETA Science Consortium International e. V, 70499 Stuttgart, Germany; PETA Science Consortium International e. V, 70499 Stuttgart, Germany

**Keywords:** new approach methodologies (NAMs), nonanimal methods, inhalation toxicity, respiratory tissue irritation, in vitro, surfactants

## Abstract

Repeated-dose exposure studies can provide insights into the toxicity of test substances, including dose–response relationships, cumulative effects, delayed responses, and reversibility of effects. A limited number of in vitro studies have assessed the effects of repeated exposure of respiratory cells to chemicals applied as liquids (via direct pipetting). The work presented in this paper builds upon these published in vitro repeated-dose studies to develop and evaluate a repeat-exposure study design for use with reconstructed human respiratory epithelium (RHRE). To this end, a bronchial RHRE (MucilAir, Epithelix) was exposed to 30 µl (91 µl/cm^2^) of 2 surfactants—oleoyl sarcosine and Triton X-100—for 4 h on up to 4 consecutive days. The results showed that the repeat-exposure protocol can be used to detect toxic effects, including differences in the responses caused by each surfactant. More specifically, while the effects of oleoyl sarcosine exposure increased over 4 d, the effects of Triton X-100 were the strongest on day 1 and decreased over the course of the experiment. This work helped refine a repeated-dose protocol that can be adapted to assess other chemistries when the effects of repeated exposure are of interest, and underscores the importance of key parameters, such as endpoint selection, timepoints, exposure duration, and washing schedules.

There has been a steady increase in the use of human cell-based models to assess the toxicity of inhaled chemicals due, in part, to well-documented differences in the anatomy and physiology between the respiratory tracts of rats and humans (Stucki et al. 2024) and an interest in increasing human relevance (van der Zalm et al. 2022; Roldan et al. 2026). In conducting a review of the PubMed database (articles up to July 15, 2024 were included) using the AI assisted web-based platform, SysRev (Bozada et al. 2021), 8 in vitro inhalation studies describing repeated exposures using liquid application of chemicals were identified (i.e. excluding particle or aerosol exposures) (Sivars et al. 2018; Xiong et al. 2018; Cervena et al. 2019; Huang et al. 2021; Tratnjek et al. 2021, 2023; OECD 2022; Sima et al. 2022). Each had differences in experimental design, including exposure volumes (10 to 40 μl), exposure frequencies and durations (ranging from single to multiple applications per day, lasting from minutes to several hours over multiple days), tissue washing protocols, and use of different in vitro model systems.

The findings highlighted an opportunity for additional research and for the development of a more standardized approach to repeated-exposure testing. Building upon the above publications and our previous work (Sharma et al. 2023; Stucki et al. 2026; US EPA 2026a, 2026b), our aim was to develop and evaluate a study design to be used when there is interest in assessing respiratory effects following repeated exposure. To this end, we used the reconstructed human respiratory epithelium (RHRE), MucilAir (Epithelix, Switzerland), a fully differentiated model of bronchial origin, that has been widely used to assess the respiratory effects of inhaled substances, including in support of regulatory decisions (McGee Hargrove et al. 2021; US EPA 2021; SCCS 2024; Boisleve et al. 2025; Roldan et al. 2026). Furthermore, pipetting was selected as an appropriate mode of exposure based on discussions with experts and existing literature (McGee Hargrove et al. 2021; US EPA 2021; SCCS 2024; Boisleve et al. 2025), including our previous study (Stucki et al. 2026). Key parameters—including washing schedule, exposure mode, timing, and duration, solvent type and temperature, and relevant endpoint selection—were carefully considered to optimize the detection of chemical-induced effects.

The detailed test protocol used in this study (see Supplementary Appendix) was reviewed by experts and their feedback was incorporated. Table S1 includes a list of materials and methods used in the study and Fig. 1 summarizes the experimental design. Briefly, MucilAir tissues (5 tissue sets; *n* = 3 per condition) were exposed to 3 different concentrations (5, 10, or 20 µg/cm^2^) of oleoyl sarcosine (CAS Number: 110-25-8) or Triton X-100 (CAS Number: 9036-19-5) for 4 h daily, on up to 4 consecutive days (30 µl or 91 µl/cm^2^). At 24 h before the first exposure, the tissues were apically washed and transepithelial electrical resistance (TEER) was measured for quality control. Only tissues with >200 Ωcm^2^ were used in the study. Subsequent re-dosing was performed only when tissues exhibited both cell viability <50% and TEER <200 Ωcm^2^ on the previous day, which was never the case in this study.

**Fig. 1.**
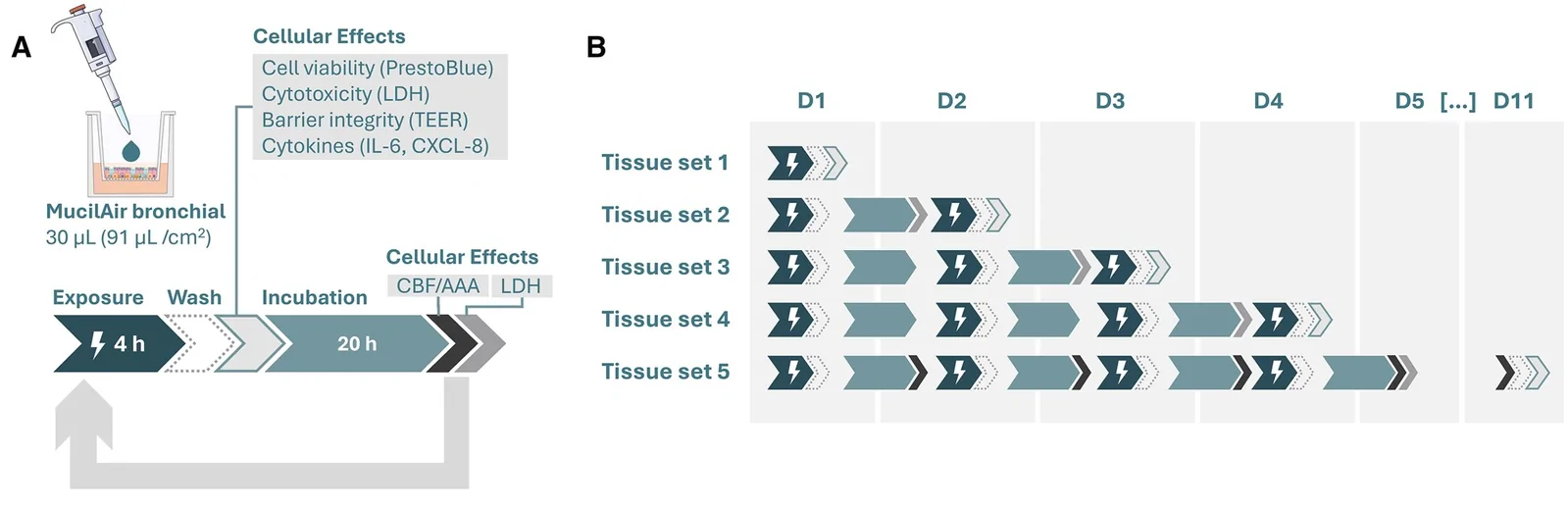
Experimental design for repeated exposure of surfactants to MucilAir tissues. A) Depicts the steps involved in each exposure. B) Illustrates the temporal layout of the experiment, including the distribution of tissue sets. AAA, average active area; CBF, cilia beating frequency; LDH, lactate dehydrogenase; TEER, transepithelial electrical resistance.

Data are presented as mean ± SD. One-way ANOVA was used to assess differences among treatment groups within each day and among study days within each treatment group. Post hoc comparisons were performed using Tukey’s honestly significant difference (HSD) test, with the vehicle control and day 1 serving as the respective reference groups.

On day 1, immediately after the first 4-h exposure, all the tissues were apically washed and 1 set of MucilAir tissues (tissue set 1) was assessed for 4 cellular effects: Cell viability (PrestoBlue), cytotoxicity (lactate dehydrogenase [LDH] release), barrier integrity (TEER), and cytokine secretion (interleukin-6 and CXCL-8), as previously described (Stucki et al. 2026) with modifications to accommodate repeat exposure time points. After the medium was refreshed for tissue sets 2 to 5, they were returned to the incubator for ∼20 h before the next exposure and collection process was repeated. This resulted in tissue sets that were treated 1, 2, 3, or 4 times with each chemical over as many days.

Tissue set 5 received 4 consecutive daily treatments, similar to tissue set 4; however, after the final exposure, instead of being immediately analyzed, the tissues were maintained in culture (with additional medium changes on days 7 and 9) and processed on day 11 to evaluate potential recovery. This was also the only tissue set for which cilia beating frequency (CBF) and average active area (AAA) were measured. On day 11, cell viability, barrier integrity, CBF (not shown), and AAA were measured to assess recovery. Cytotoxicity and cytokine secretion could not be assessed on day 11 because the medium was refreshed on intermittent days to maintain tissues in the incubator.

Cell viability was assessed immediately after the 4-h exposure, and no change was observed for tissues treated with 5 or 10 µg/cm^2^ of either surfactant on any day. For tissues treated with 20 µg/cm^2^ oleoyl sarcosine, a significant decrease in cell viability was observed on days 3 and 4 (Fig. 2A) with no recovery on day 11 (Fig. S1A). Treatment with 20 µg/cm^2^ Triton X-100 led to a significant decrease in cell viability on days 1 and 4 (Fig. 2B), with tissues showing recovery to normal levels at day 11 (Fig. S1B).

**Fig. 2.**
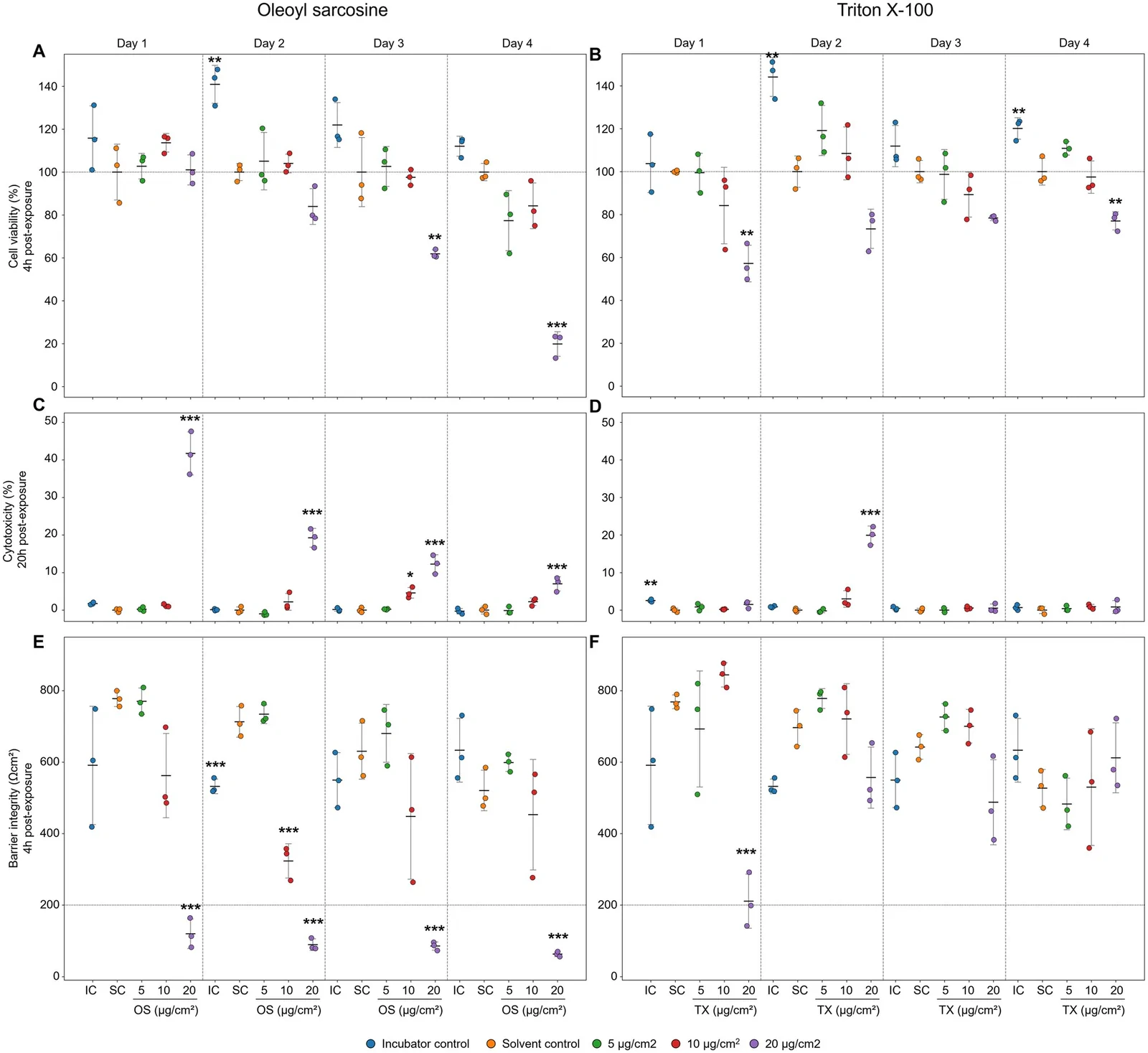
Determination of cell health after repeated exposure to surfactants. Four sets of MucilAir tissues (with 1 set for each day, including all treatment conditions represented in different colors) were exposed to 30 µl (91 µl/cm^2^) of 3 different concentrations (5,10, or 20 µg/cm^2^) of oleoyl sarcosine or Triton X-100, for 4 h per day for up to 4 consecutive days. A) and B) show percent cell viability determined using a resazurin-based assay (PrestoBlue) immediately after the 4 h of exposure per day. C) and D) show cytotoxicity determined using LDH release measured 20 h after exposure expressed as percentage. E) and F) show barrier integrity (TEER values in Ωcm^2^) measured after 4 h of exposure each day. The asterisks (*) show statistical significance compared with solvent control: *P < 0.05; **P < 0.01; ***P < 0.001.

Cytotoxicity was measured at 2 time points: Immediately after the 4-h exposure (Fig. S2) and ∼20 h after the exposure (directly before the next exposure; Fig. 2C and D). In general, the LDH release measured immediately after the 4-h exposure showed similar trends to those observed after the 20-h postexposure incubation, although the effects were less pronounced.

No cytotoxicity compared with solvent controls was observed 20 h after exposure to 5 or 10 µg/cm^2^ oleoyl sarcosine on any day, with the exception of a minor but statistically significant increase in cytotoxicity following exposure to 10 µg/cm^2^ oleoyl sarcosine on day 3 (Fig. 2C). When exposed to 20 µg/cm^2^ oleoyl sarcosine, a statistically significant increase in cytotoxicity was observed 20 h postexposure compared with solvent controls on all days. Cytotoxicity peaked on day 1 and declined daily, though results were not normalized to cell count, which may explain the daily decrease. No cytotoxicity was observed after exposure to 5 or 10 µg/cm^2^ Triton X-100 on any day. An increase in cytotoxicity was only observed in tissues exposed to 20 µg/cm^2^ Triton X-100 on day 2 (Fig. 2D).

Barrier integrity was assessed using the TEER assay immediately after the 4-h exposure, following the reporting recommendations set by Sharma et al. (2025). Using a common threshold to determine barrier integrity of MucilAir tissues (200 Ωcm^2^), TEER values remained above this threshold on all days in tissues treated with 5 or 10 µg/cm^2^ of oleoyl sarcosine or Triton X-100 (Fig. 2E and F, respectively). On day 2, a statistically significant decrease that remained well above 200 Ωcm^2^ was observed for tissues treated with 10 µg/cm^2^ oleoyl sarcosine. For tissues treated with 20 µg/cm^2^ oleoyl sarcosine, the TEER value dropped and remained below 200 Ωcm^2^ on all days, including day 11 (Fig. 2E and Fig. S1C). In contrast, tissues treated with 20 µg/cm^2^ Triton X-100 remained above 200 Ωcm^2^ each day, including day 11 (but dropped significantly, to almost 200 Ωcm^2^, on day 1) (Fig. 2F and Fig. S1D).

For tissues treated at the highest concentration (20 µg/cm^2^) of oleoyl sarcosine, cell viability and cytotoxicity decreased more with each day, and barrier integrity remained low across all time points. Normally, cell viability and cytotoxicity are inversely correlated (i.e. when cell viability decreases, cytotoxicity increases). One possible explanation is that repeated exposures progressively reduce the number of viable cells, which reduces the overall amount of LDH available for release in the remaining cells each day, eventually leading to a complete destruction of the tissues, as seen in low TEER values on day 11. In contrast, tissues exposed to 20 µg/cm^2^ Triton X-100 showed reduced cell viability and barrier integrity, but no increase in cytotoxicity on day 1. Cell viability remained below the solvent control (although not always statistically significant) but seemed to increase over the exposure days (58% cell viability on day 1 vs 78% on day 4). Because this reduction was not accompanied by increased cytotoxicity (except on day 2), it may reflect altered cellular metabolic activity rather than cell death. Also, barrier integrity recovered after day 1. Together, these observations suggest that the tissues may have partially recovered during repeated exposures. Daily micrographs or histology would have helped confirm these hypotheses.

Cytokine release (IL-6 and CXCL-8) and cilia metrics (CBF and AAA) were also evaluated in this study and followed similar trends as the other cellular effects (Figs S1E and F, S3, and S4). For the sake of brevity, they are not discussed further.

Our results demonstrate that a single 4-h exposure to the lowest concentrations (5 and 10 µg/cm^2^) of either surfactant did not affect cell viability, cytotoxicity, or barrier integrity. These lower concentrations were included to evaluate whether repeated exposures produced cumulative effects. However, no evidence of such effects was observed.

One objective of this work was to evaluate whether repeated-exposure testing provides additional information beyond single-exposure testing for these surfactants. A 4-h exposure was used for this study (instead of a 24-h exposure that was used in our previous study; Stucki et al. 2026) to avoid a semisubmerged culture over 4 d of treatment. Comparisons with our previous 24-h single-exposure study (Stucki et al. 2026) should be interpreted cautiously, as only one repeat-exposure experiment was conducted. Nevertheless, the 4-h observations in this repeated-dose study aligned with some of our previous 24-h single exposure observations in that the 2 lowest concentrations showed no effect and the 20 µg/cm^2^ concentration produced substance-specific responses. Oleoyl sarcosine elicited comparable reductions in barrier integrity and increases in cytotoxicity following both 4- and 24-h exposures. Triton X-100 also caused a similar reduction in barrier integrity for both exposure durations, but differed slightly in cell viability and cytotoxicity. Overall, these observations indicate that low concentrations of surfactants did not affect cell health, whereas higher concentrations revealed substance-specific cellular responses. Surfactants act through rapid physical interactions with cellular membranes, possibly explaining why no major differences were observed between the 4- and 24-h exposure durations for these substances.

For a direct comparison of 4-h single, 24-h single, and 4 × 4-h repeat exposure, ideally, the studies would be conducted with higher concentrations/broader concentration range, have more experimental runs, and would include daily micrographs or histology. Despite these limitations, our results suggest that, for these 2 surfactants, repeated-dose testing did not provide substantial additional information beyond single-exposure testing. Therefore, the additional complexity, time, and resource requirements associated with repeated-dose testing may not be justified for rapidly acting surfactants when robust single-exposure data already exist.

The experimental plan for this study was developed with careful consideration of factors such as number, duration, and mode of exposures; selection of relevant biological markers and time points for assessment; and the inclusion of tissue-washing steps at appropriate intervals. Although the exposure duration (4 vs 24 h) did not seem to effect these results, for future studies, assay kinetics (i.e. the incubation time before measuring cellular effects) should be considered to optimize detection windows (e.g. LDH release, cytokine production, and effects on AAA require time to manifest and may not be measurable at 4 h). A future study could begin with baseline measurements and quality control on Friday, allowing the first exposure to occur on Monday and enabling flexible postexposure sampling during the work week. Also, to save resources, when repeated-dose testing is of interest, one could assess the cellular endpoints only on days 1 and 4 rather than all 4 d. In addition, histology could be included depending on the goal of testing. Histology was included in this study, however, due to logistical issues, the tissues were not processed promptly after fixation (i.e. within 2 wk of receiving at the facility) and were therefore unusable.

Considering the paucity of data and varying protocols that have been used for in vitro repeated dose exposure studies using liquid application of test chemicals, this study built upon previously published repeat-exposure studies to optimize and evaluate a protocol to use for in vitro repeated dose testing of surfactants. The resulting protocol, provided in the Supplementary Appendix is described in sufficient detail to facilitate its application and further evaluation by other researchers. Our findings highlight the importance of understanding the properties of the test chemicals and kinetics of toxicity (i.e. timepoints to assess) and suggest that no exposure scenario (single vs repeat dose) is universally optimal. The 2 surfactants tested here are direct-acting irritants that act rapidly through physical interactions with the cellular membrane. This may explain why repeated exposure did not produce substantially different responses from those observed following single exposure and therefore did not provide added value for testing the 2 surfactants. For testing different chemical classes, careful consideration of the mode of toxicity and purpose of testing is recommended to determine whether the additional time, cost, and logistical effort required for repeat-dose protocols are justified by a meaningful increase in sensitivity or predictive value.

## Supplementary Material

kfag146_Supplementary_Data
