# Supplementary material for: Human in vitro airway models to assess point-of-contact respiratory toxicity of surfactants: development and evaluation of a repeated-exposure study design
Source: Toxicol Sci. 2026 Oct 30;209(11):kfag146. doi: 10.1093/toxsci/kfag146 (PMC13769445; doi:10.1093/toxsci/kfag146)
Supplement: Supplementary file 1 [file kfag146_supplementary_data.docx]

**Supplementary Document**

**Human in vitro airway models to assess point-of-contact respiratory toxicity of surfactants: development and evaluation of a repeated-exposure study design**

Monita Sharma^1^<https://orcid.org/0000-0003-2859-2581>; Andreas O. Stucki^1^<https://orcid.org/0000-0002-0997-8502>; Sandra Verstraelen^2^<https://orcid.org/0000-0003-0529-8208>; Karen Hollanders^2^; An Jacobs^2^; Nuria Roldan^1^<https://orcid.org/0000-0002-3728-804X>; Sylvie Remy^2^<https://orcid.org/0000-0003-0322-9620>; Adam Bettman^1^; Amy J. Clippinger^1^<https://orcid.org/0000-0003-3623-1196>

^1^PETA Science Consortium International e.V., 70499 Stuttgart, Germany.

^2^Environmental Intelligence Unit, Flemish Institute for Technological Research (VITO), 2400 Mol, Belgium.

*Corresponding author: Monita Sharma ([monitas@thepsci.eu](mailto:monitas@thepsci.eu))

PETA Science Consortium International e.V.
Friolzheimer Str. 3
70499 Stuttgart
Germany

# Supplementary Figures

**Supplementary Figure 1. Assessment of cellular effects at 7 days of recovery after the last of four exposures (Day 11).** The graphs show cell viability (A-B), barrier integrity (C-D), and average active area (AAA; E-F)) for one set of tissues (Set 5) exposed to three different concentrations (5,10, or 20 µg/cm^2^) of oleoyl sarcosine (A, C, E) or Triton X-100 (B, D, F), for four hours per day for four consecutive days followed by recovery of 7 days after last exposure. The asterisks (*) show statistical significance compared to solvent control: *, *p*<0.05; **, *p*<0.01; ***, *p*<0.001.

**Supplementary Figure 2. Determination of cytotoxicity after repeated exposure to surfactants (assessed immediately after the last 4-hour exposure of each day).** Four sets of MucilAir tissues (with one set for each day, including all treatment conditions) were exposed to three different concentrations (5, 10, or 20 µg/cm^2^) of oleoyl sarcosine (A) or Triton X-100 (B), for four hours per day for four consecutive days. Figures show cytotoxicity measured immediately after 4-hours of exposure using LDH release expressed as fold change compared to solvent control. The asterisks (*) show statistical significance compared to solvent control: *, *p*<0.05; **, *p*<0.01; ***, *p*<0.001.

**Supplementary Figure 3. Assessment of cytokine secretion after repeated exposure to surfactants (immediately after the last 4-hour exposure of each day).** Four sets of MucilAir tissues (with one set for each day, including all treatment conditions) were exposed to three different concentrations (5,10, or 20 µg/cm^2^) of oleoyl sarcosine (A and C) or Triton X-100 (B and D), for four hours per day for four consecutive days. Samples from the basolateral medium were collected to measure secreted Interleukin (IL)-6 and CXCL-8 immediately following 4hour exposure to oleoyl sarcosine (A and B) or Triton X-100 (C and D). The asterisks (*) show statistical significance compared to solvent control: *, *p*<0.05; **, *p*<0.01; ***, *p*<0.001.

**Supplementary Figure 4. Assessment of cilia beat frequency (CBF) and average active area (AAA) in bronchial MucilAir tissues ~20 hours post-exposure.** Figures show CBF (A and B) and AAA (C and D) measurements of MucilAir tissue Set 5 exposed to three different concentrations (5,10, or 20 µg/cm^2^) of oleoyl sarcosine (A and C, respectively) or Triton X-100 (B and D, respectively), for four hours per day for up to four consecutive days. CBF is presented in hertz (Hz) and AAA as percentage (%) of the examined regions. The asterisks (*) show statistical significance compared to solvent control: *, *p*<0.05; **, *p*<0.01; ***, *p*<0.001.

# Supplementary Table

**Supplementary Table 1. List of materials used in this study.**

| **Product name** | **Source/Manufacturer** | **Catalog #** |
| --- | --- | --- |
| Basler acA1300-200µm USB3 video camera | Ammons Engineering, Clio, Michigan, USA | n/a |
| Calcium chloride dihydrate | Merck Production Chemicals, Darmstadt, Germany | 1.42002.1000 |
| Caps for glass vials | Thermo Fisher Scientific, Waltham, Massachusetts, USA | 9150868 |
| Cell culture multi-well plate, 24-well, polystyrene, clear | Greiner Bio-One nv, Kremsmünster, Austria | 662160 |
| Cilia-X software | Epithelix Sàrl, Geneva, Switzerland | CiliaX V1.0 |
| Cytotoxicity LDH Assay Kit-WST | Tebu-bio, Le Perray-en-Yvelines, France | Ck12-05 |
| Dimethyl sulfoxide (DMSO) | Sigma-Aldrich, Saint Louis, Missouri, USA | D5879 |
| EVOM Manual voltohmmeter | World Precision Instruments Germany, GmbH, Friedberg, Germany | EVM-MT-03-01 |
| EVOM STX-4 electrodes | World Precision Instruments Germany, GmbH, Friedberg, Germany | STX4 |
| Formaldehyde 4% | Avantor, Radnor, Pennsylvania, USA | 9713 |
| Gibco DPBS, calcium, magnesium | Thermo Fisher Scientific, Waltham, Massachusetts, USA | 11580456 |
| Gibco HEPES (1M) | Thermo Fisher Scientific, Waltham, Massachusetts, USA | 15630080 |
| Glass vials | Thermo Fisher Scientific, Waltham, Massachusetts, USA | 11090500 |
| Lipopolysaccharides (LPS) from Escherichia coli 0111:B4 | Sigma-Aldrich, Saint Louis, Missouri, USA | L4391 |
| Methanol (MeOH) | Sigma-Aldrich, Saint Louis, Missouri, USA | 1.06018 |
| Microplate, 96 well, PS, F-bottom (chimney well) | Greiner Bio-One nv, Kremsmünster, Austria | 655180 |
| MilliQ water | Merck KGaA, Darmstadt, Germany | n/a |
| MucilAir | Epithelix Sàrl, Geneva, Switzerland | EP01MD |
| MucilAir Medium | Epithelix Sàrl, Geneva, Switzerland | EP05MM |
| Multi-mode microplate reader CLARIOstar Plus | BMG Labtech, Ortenberg, Germany | n/a CLARIOstar V5.21 |
| Oleoyl sarcosine | Tokyo Chemical Industry (TCI) Europe N.V., Zwijndrecht, Belgium | O0113 (Lot: EEY8F-RK) |
| PrestoBlue Cell Viability Reagent | Thermo Fisher Scientific, Waltham, Massachusetts, USA | A13261 |
| Screw cap tubes (25 mL) | Eppendorf, Hamburg, Germany | 30122437 |
| Sealing Tape | Nunc, Sjaelland, Denmark | 236366 |
| Sisson-Ammons Video Analysis (SAVA) software | Ammons Engineering, Clio, Michigan, USA | n/a |
| Sodium chloride | Sigma-Aldrich, Saint Louis, Missouri, USA | S7653 |
| Triton X-100 | Sigma-Aldrich, Saint Louis, Missouri, USA | X100 (Lot: WXBD9346V) |
| V-PLEX Custom Human Biomarkers Proinflammatory Panel (human): Human IL-6, Human IL-8 | Meso Scale Diagnostics, Rockville, Maryland, USA | K151A9H-2 |
| Zeiss Axiovert 10 microscope | Zeiss, Oberkochen, Germany | n/a |

# Supplementary Appendix: Study protocol

The sections below include the detailed stepwise protocol used in this study. In this study, a buffered saline solution was used as vehicle control. However, other vehicles, including MucilAir medium, PBS, non-buffered saline, or water, may be appropriate depending on the test substance and study objectives. The protocol described here reflects the conditions used in the present study and may require adaptation for other applications. Certain steps may also differ from recommendations provided by the tissue supplier. Users should carefully evaluate the suitability of the protocol, including vehicle selection and exposure conditions, before applying it in a different experimental context.

## 1 MucilAir reception and maintenance before exposure

- Tissue Model: MucilAir-Bronchial.
- Start the experiment within one week of the reception of tissues.
- Unless specified, all steps in this protocol must be conducted under sterile working conditions using aseptic techniques to prevent contamination of the tissue cultures.
- Incubator conditions used for the entire study: 37°C (± 2°C), 5% CO_2_ (± 1%); ≥95% relative humidity.
- Document any deviation from this protocol.

### 1.1 Day –6 or –5: MucilAir are received and put into culture (*Tuesday or Wednesday*)

- Warm MucilAir Medium to 37°C.
- Add 700 µL of warm MucilAir Medium to each well of a 24-well plate.
- Transfer inserts into the pre-filled 24-well plate using sterilized forceps.
- Put the plate into the incubator at 37°C.

### 1.2 Day –3: MucilAir are washed and medium is refreshed (*Friday*)

- Warm MucilAir Medium to 37°C.
- Transfer the plates with the tissue inserts to the laminar flow cabinet.
- Add 200 µL of warm MucilAir Medium to the apical side of each insert.
- Put the plates with the tissue inserts back in the incubator for 5 min at 37°C.
- Gently pipet medium up and down two times with 100 µL pipette to resuspend any mucus present.
- Gently aspirate medium by slightly tilting the plate and aspirating from the side of the insert and well (apically and basally).
- Add 700 µL of warm MucilAir Medium basally and return the plates with tissue inserts to the incubator.

### 1.3 Day 0: MucilAir pre-exposure steps (*Monday, all tissue sets*)

- Warm MucilAir Medium to 37°C.
- Transfer the plates with the tissue inserts corresponding to tissue Set 5 to the microscope to measure CBF and AAA.
- Transfer all the plates with tissue inserts to the laminar flow cabinet.
- Add 200 µL of warm MucilAir Medium to the apical side of each insert.
- Put the plates with the tissue inserts back in the incubator for 5 min at 37°C.
- Transfer the cells to the laminar flow cabinet to measure TEER pre-dosing. Record the time when they are taken out of the incubator.
- Measure TEER (for baseline measurements and quality control (QC); healthy tissues are characterized by a TEER of 200 Ω cm^2^ or higher. Tissues below 200 Ω cm^2^ must not be used. For each plate, record the time when the last insert of the plate was measured.
- Gently pipet apical medium up and down two times with 100 µL pipette to resuspend any mucus present.
- Gently aspirate medium by slightly tilting the plate and aspirating from the side of the insert and well (apically and basally).
- Add 700 µL of warm MucilAir Medium basally and return the plates to the incubator at 37ºC until day 1 (*i.e.,* exposure).
- Prepare saline solution ahead of time to be used on day 1.

## 2 MucilAir exposure (*Monday*)

### 2.1 Range-finding and main study

- A range-finding study will be conducted to inform the selection of test concentrations for the main study and will include a single 4-hour exposure and assessment of barrier function, cell viability, and cytotoxicity (TEER assay, the resazurin-based PrestoBlue assay, and LDH release). To do so, follow the current protocol up to day 1 followed by the endpoints specified above. No chemical analysis or CBF will be done for range finding. The number of necessary inserts for dose range-finding and main study can be found in Appendix Table 1 and 2, respectively.

**Appendix Table 1: Test conditions and number of tissue inserts for range-finding study.**

| Incubator controls (IC) | 2 inserts | 2 |
| --- | --- | --- |
| Solvent control (SC) OS | 2 inserts | 2 |
| SC Triton X-100 | 2 inserts | 2 |
| LDH control from kit | 1 insert | 1 |
| OS: 5 test concentrations | 2 inserts | 10 |
| Triton X-100: 5 test concentrations | 2 inserts | 10 |
| **Total number of inserts per run** | | **27** |

**Appendix Table 2: Test conditions and number of inserts for main study.**

|  | **Replicates per tissue set** | **Tissue Sets*** | **#Total inserts** |
| --- | --- | --- | --- |
| Incubator controls (IC) | 3 inserts | 5 | 15 |
| Solvent control (SC) OS | 3 inserts | 5 | 15 |
| SC Triton X-100 | 3 inserts | 5 | 15 |
| LDH control from kit | 1 insert | 5 | 5 |
| Lipopolysaccharide (LPS) | 1 insert | 5 | 5 |
| OS: 3 test concentrations | 3 inserts | 5 | 45 |
| Triton X-100: 3 test concentrations | 3 inserts | 5 | 45 |
| **Total number of inserts per run** | | | **145** |

*Each tissue set includes all tissues (covering all test conditions) investigated after each exposure (1-4 days and 7 days post-4-day exposure). For 4 days of exposures, there are 4 tissue sets (one set for each day). One additional tissue set (Set 5) is kept for an additional 7 days after a 4-day exposure to investigate recovery.

### 2.2 Exposure scheme

- Repeated dosing will be conducted as depicted in **Appendix Table 3.**
- One tissue set is processed each day. The rest of tissue sets continue repeated exposure according to the exposure scheme. For concentrations scheduled for re-dosing, continuation was evaluated separately for each test concentration. Re-dosing of a concentration in the remaining tissue sets was to be discontinued only if all three replicates showed both cell viability <50% and TEER <200 Ω·cm² on two consecutive assessment days.

**Appendix Table 3. Scheme for repeat dose testing and recovery.** *The time proposal in the first column is hypothetical and intended to show the tentative timeline of experiments.

|  | **REPEATED EXPOSURE** | | | | | **RECOVERY** | | | |
| --- | --- | --- | --- | --- | --- | --- | --- | --- | --- |
| **Time*** | **Day 0** | **Day 1** | **Day 2** | **Day 3** | **Day 4** | **Day 5** | **Day 7** | **Day 9** | **Day 11** |
|  |  | **20±1 hours since MC1** | **20±1 hours since MC2** | **20±1 hours since MC3** | **20±1 hours since MC4** | **20±1 hours since MC5** | **44±1 hours after MC6** | **44±1 hours after MC7** | **44±1 hours after MC8** |
| 07:30 | Set 5 - CBF/ AAA | Set 5 – CBF/AAA | Set 5 – CBF/AAA | Set 5 – CBF/AAA | Set 5 – CBF/AAA | Set 5 - CBF/AAA |  |  | Set 5 - CBF/AAA |
| 08:00 |  | Set 1 – collect basal medium for LDH | Set 2 – collect basal medium for LDH | Set 3 – collect basal medium for LDH | Set 4 – collect basal medium for LDH | Set 5 – collect basal medium for LDH |  |  |  |
|  | All sets – TEER Re-establish ALI **(Medium Change (MC)1)** | All sets – Fresh medium basal | All sets – Fresh medium basal | All sets – Fresh medium basal | All sets – Fresh medium basal | Set 5 - Fresh medium basal, re-establish ALI **(MC6)** |  |  |  |
| 09:00 | Tissues outside of the quality control range (200 Ω cm^2^) must not be used for testing | Exposure (4h) - Sets 1-5 | Exposure (4h) - Sets 2-5 | Exposure (4h) - Sets 3-5 | Exposure (4h) - Sets 4-5 |  |  |  | Exposure (4h) - ONLY positive controls (LDH, LPS) |
| 13:00 |  | Set 1 – Collect basal and apical sample  Sets 1-5- Wash | Set 2 – Collect basal and apical sample  Sets 2-5 - Wash | Set 3 – Collect basal and apical sample  Sets 3-5 - Wash | Set 4 – Collect basal and apical sample  Sets 4-5 - Wash |  | Set 5 - Wash | Set 5 - Wash | Set 5 – Collect basal and apical sample followed by wash |
|  |  | Set 2, 3, 4, 5 – Fresh medium basal, re-establish ALI **(MC2)** | Set 3, 4, 5 - Fresh medium basal, re-establish ALI **(MC3)** | Set 4, 5 - Fresh medium basal, re-establish ALI **(MC4)** | Set 5 - Fresh medium basal, re-establish ALI **(MC5)** |  | Set 5 - Fresh medium basal, re-establish ALI **(MC7)** | Set 5 - Fresh medium basal, re-establish ALI **(MC8)** |  |
|  |  | Set 1 - Measure TEER, LDH, Cytokines, PrestoBlue, Histology | Set 2 - Measure TEER, LDH, Cytokines, PrestoBlue, Histology | Set 3 - Measure TEER, LDH, Cytokines, PrestoBlue, Histology | Set 4  Measure TEER, LDH, Cytokines, PrestoBlue, Histology | Set 5 -  Measure LDH |  |  | Set 5 -  Measure TEER, LDH, Cytokines, PrestoBlue, Histology |
|  |  | If cell viability is <50% (PrestoBlue) on previous day and TEER < 200 Ω cm^2^, do not use the tissues for further testing | | | | |  |  | Report data from TEER, LDH, and PrestoBlue |

### 2.3 Test concentrations

- The concentrations to be used for the dose-range finding and the main studies can be found below in **Appendix Tables 4 and 5**, respectively. Chemical analysis is to be performed every day for each test chemical concentration (See section 2.5)

**Appendix Table 4: Concentrations for the range-finding study.**

| **Oleoyl sarcosine** | | | | **Triton X-100** | | | |
| --- | --- | --- | --- | --- | --- | --- | --- |
| **µM** | **µL** | **cm^2^** | **µg/cm²** | **%** | **µL** | **cm^2^** | **µg/cm²** |
| 2489 | 30 | 0.33 | 80 | 0.0822 | 30 | 0.33 | 80 |
| 1244.5 | 30 | 0.33 | 40 | 0.0411 | 30 | 0.33 | 40 |
| 622.26 | 30 | 0.33 | 20 | 0.0206 | 30 | 0.33 | 20 |
| 311.125 | 30 | 0.33 | 10 | 0.0103 | 30 | 0.33 | 10 |
| 155.56 | 30 | 0.33 | 5 | 0.0051 | 30 | 0.33 | 5 |

**Appendix Table 5: Concentrations for main study.**

| **Oleoyl sarcosine** | | | | **Triton X-100** | | | |
| --- | --- | --- | --- | --- | --- | --- | --- |
| **µM** | **µL** | **cm^2^** | **µg/cm²** | **%** | **µL** | **cm^2^** | **µg/cm²** |
| 622.26 | 30 | 0.33 | 20 | 0.0206 | 30 | 0.33 | 20 |
| 311.125 | 30 | 0.33 | 10 | 0.0103 | 30 | 0.33 | 10 |
| 155.56 | 30 | 0.33 | 5 | 0.0051 | 30 | 0.33 | 5 |

### 2.4 Test chemicals and controls preparation (Day 1-4, except saline solution at D0)

- Warm MucilAir Medium and saline solution to 37°C.
  - Prepare all test chemicals and controls as described below.

#### 2.4.1 Saline solution (buffer)*

- On day 0, prepare a fresh batch of buffer by combining 0.9 % (w/v) sodium chloride (NaCl, weigh 4.5 g NaCl and add water to final volume of 500 mL) + 1.25 mM calcium chloride (CaCl_2_, weigh 1.47 g CaCl_2_ and add 10 mL water) + 10 mM HEPES: 100 ml = 125 µL 1M CaCl_2_ + 1 mL 1M HEPES + 98.875 mL 0.9 % (w/v) NaCl. Sterile filter it, label the container with the date of preparation, and store at 4ºC. Use for up to four weeks.
- Note: Other vehicle controls than saline solution might be used such as MucilAir medium, PBS, non-buffered saline, water, etc.

#### 2.4.2 Oleoyl sarcosine

- SC = 0.5% (v/v) dimethyl sulfoxide (DMSO) in saline solution.
- Prepare oleoyl sarcosine stock and working concentrations freshly before each exposure.
- Preparation of Oleoyl sarcosine stock and working concentrations:
- Make a stock of 0.4978 M oleoyl sarcosine in 100% DMSO by pipetting from the neat Oleoyl sarcosine (liquid) using a positive displacement pipette. Use sterilized glass vials Fisherbrand Cat.No. 10000782, screw vial 20 mL EPA clear. From the 0.4978 M stock, make a 2-fold serial dilution in 100% DMSO in the same glass vials. Mix by vortexing.
- From the serial DMSO dilutions, make a 200x dilution in saline buffer (0.9% Natrium chloride (NaCl) + 1.25 mM calcium chloride (CaCl_2_) + 10 mM HEPES). Mix by vortexing.
- Finally prepare the solvent control (SC), by dissolving 0.5% DMSO in saline solution.

#### 2.4.3 Triton X-100

- SC = saline solution
  - Prepare Triton X-100 stock and working concentrations freshly before each exposure.
  - Preparation of Triton X-100 stock and working concentrations:
  - Make a 0.0822 % (v/v) stock solution of Triton X-100 in saline buffer (0.9% Natrium chloride (NaCl) + 1.25 mM calcium chloride (CaCl_2_) + 10 mM HEPES) from the neat Triton X-100 (liquid) by using a positive displacement pipette. Use sterilized glass vials Fisherbrand Cat.No. 10000782, screw vial 20 mL EPA clear.
  - From the concentrated stock, make 2-fold serial dilutions in the corresponding vehicle using the same glass vials. Mix by vortexing.
  - Finally prepare the solvent control (SC), which is saline buffer in this case.

#### 2.4.4 LPS (Escherichia coli, O111: B4)

- Pro-inflammatory positive control.
- Prepare LPS (1 mg/ml) stock as indicated below:
- Make a stock of 1 mg/mL LPS in PBS by resuspending the whole powder (1 mg) in 1 mL PBS in the original vial of LPS. Mix by gently pipetting.
- Aliquots of 20 µL are prepared in Eppendorf tubes (1.5 mL). Aliquots are stored at -20°C.

***Do not freeze-thaw the aliquots. After defrosting and using an aliquot, do not store remaining volume.***

- Dilute stock LPS 1 mg/ml (–20°C) 1/5 in saline solution (final concentration 200 µg/mL) and mix by pipetting. Prepare freshly before each exposure.

#### 2.4.5 LDH release

- LDH positive control from kit.

### 2.5 Chemical analysis of test chemical working concentrations

- Prepare a glass vial for each concentration of test chemical for chemical analysis:
- Dilute 1/10 as follows: 500 µL methanol (MeOH) + 100 µL sample + 400 µL MilliQ water.
- Samples are further diluted 10x or 100x (to fit the calibration curve) for liquid chromatography and mass spectrometry (LC-MS/MS) analysis.

### 2.6 CBF/AAA measurement (day 1-4, tissue 5 only, pre-dosing)

- Transfer the tissue inserts from **Set 5 only** to the stage-top incubator to conduct CBF/AAA measurements. Use the Cilia-X software to measure and analyze CBF/AAA.
- Transfer the plates with the tissue inserts to the laminar flow cabinet for dosing.

### 2.7 Exposure to test chemicals/controls (Day 1-4, all tissue sets)

- Transfer the plates with the tissue inserts (**all sets**) to the laminar flow cabinet for dosing. Do this sequentially to avoid having the tissues too long out of the incubator.
- Transfer the inserts from the set that will be analyzed that day (*i.e.,* Set 1 on day 1, Set 2 on day 2, etc.) to a new plate with fresh basal medium. Store the old plates containing basal medium (for the tissue set that will be analyzed that day) at 4ºC for LDH measurement together with the post-exposure samples.
- Aspirate basal media for all remaining tissues.
- Add 700 µL of warm MucilAir Medium basally.
- Apply apically 30 µL of test chemical (n=3 tissues/concentration) or controls (n=3 tissues/solvent controls; n=1 tissue for LPS, n=1 for LDH), using a positive displacement or air displacement pipette, taking care not to touch the tissue surface with the tip. Ensure that tissues are homogeneously covered with liquid by gently tapping the side of the plate. Note the time of dosing on each plate.
- In parallel, an untreated set of controls (IC) undergoes the same processes and sampling without a dose applied.
- Return the plates with the tissue inserts to the incubator for 4 hours (± 10 min).
- At completion of exposure time, for the corresponding tissue set to be investigated, follow Section 3: Exposure termination and assays.
- Process remaining tissue sets to be re-dosed or for recovery.

### 2.8 Washing and re-establishment of ALI 4 hours after exposure (Day 1-4, corresponding Sets)

- Warm MucilAir Medium and saline solution to 37°C
- Wash the apical surface three times with saline solution:
  - First wash:
    - Add 200 µL saline solution to the apical side of the tissue inserts and transfer the plate with the tissue inserts back to the incubator for 5 min at 37°C.
    - After 5 min, transfer the plate with the tissue inserts back to the laminar flow cabinet.
    - Use a 100 µL pipette and gently pipette three times up and down.
  - Gently remove the apical saline solution using a pipette and discard the aspirate. Second wash and third wash:
    - Add 200 µL saline solution to the apical side of the tissue inserts. Use a 100 µL pipette and gently pipette three times up and down. Remove the apical saline solution using a pipette and discard.
- Using sterilized forceps, place tissues in a new plate with 700 µL warm MucilAir Medium.
- Transfer the plates with tissue inserts back to incubator.
- For repeated exposure (days 1-3 for corresponding tissue sets) see section 2.7. For recovery (only on day 4 for tissue Set 5), follow sections 4 and 5.

### 2.9 Decision criteria for continuation/stopping of experiment

- For tissues of certain working concentrations to move forward with re-dosing, they must:
  - Have a viability >50% (PrestoBlue) and TEER ≥ 200 Ω cm^2^ for 2 days consecutively.
  - If all three replicates of this working concentration fail to comply with these conditions, re-dosing will be aborted for that concentration(s) for the remaining tissue sets.

### 2.10 Re-exposure to test chemicals/controls on the following day (Day 2-4, corresponding sets)

- Repeat steps for exposure and following procedures.

## 3 Exposure termination and assays

### 3.1 Preparation of PrestoBlue solution

- Prepare a 10 % (v/v) solution of PrestoBlue in MucilAir Medium. Keep it in dark or wrap in aluminum.

### 3.2 After 4-hour exposure (Day 1-4, corresponding tissue Set)

#### 3.2.6 Medium collection and apical wash

- Warm MucilAir Medium, PrestoBlue solution, and saline solution to 37°C.
- Prepare a new 24-well plate with 700 µL warm MucilAir Medium.
- Transfer the plates with the tissue inserts to the laminar flow cabinet.
- Using sterilized forceps, place the tissue inserts in the new plate with 700 µL warm MucilAir Medium.
- Store the old plate with basal medium at 4°C (ideally no longer than 4 hours) until further processing for LDH analysis and cytokines.
- Wash the apical surface two times with saline solution to remove the test chemical:
  - First wash:
    - Add 250 µL saline solution to the apical side of the tissue inserts and transfer the plate with the tissue inserts back to the incubator for 5 min at 37°C.
    - After 5 min, transfer the plate with the tissue inserts back to the laminar flow cabinet.
    - Use a 100 µL pipette and gently pipette up and down three times. Collect a total volume of 200 µL in tube or 96-well plate.
    - Centrifuge for 300g 5 min to remove cells and debris. Collect the supernatant and store at –80°C (for apical cytokines). Store a sample of saline solution to serve as a baseline.
  - Second wash:
    - Add 200 µL saline solution to the apical side of the tissue inserts. Use a 100 µL pipette and gently pipette three times up and down. Remove the apical saline solution using a pipette and discard.

#### 3.2.7 TEER

- Warm MucilAir Medium to 37°C.
- Add 200 µL warm MucilAir medium apically.
- Transfer the plates with tissue inserts to the incubator and incubate at 37°C for 5 min.
- Transfer the plates with the tissue inserts to the laminar flow cabinet to measure TEER. Record the time each plate is removed from the incubator.
- Measure TEER. Record the time when the last insert of the plate was measured.
- Gently pipette the apical solution up and down two times with a 100 µL pipette.
- Gently remove the apical medium by slightly tilting the plate and pipetting from the side of the insert.

#### 3.2.8 Cell viability: PrestoBlue

- Using the same plate with tissue inserts from the previous step (including the one insert for LDH and LPS), add 200 µL of 10% (v/v) PrestoBlue in warm MucilAir Medium apically in each insert.
- Incubate the tissue inserts and 10% PrestoBlue solution (for the blank controls) for 1 hour at 37°C.
- From each insert, transfer duplicates (2x 90 µL apically) into a 96-well plate.
- Add 2x 90 µL of 10% (v/v) PrestoBlue in MucilAir Medium (*i.e.*, blank controls) to the 96-well plate.
- Measure fluorescence excitation 560 /emission 610 nm on a multi-plate reader.
- Add the raw values to the excel file for automatic calculations of results

#### 3.2.9 Cytotoxicity: LDH assay

- Take plate with old basal medium samples and from each well, transfer 2x 100 µL sample to a 96-well plate (duplicates). The sample from the LDH positive control insert should be diluted appropriately in MucilAir Medium.
- Add 2x 100 µL MucilAir medium as blank to an additional well in the 96-well plate.
- Add 100 µL LDH substrate mix (working solution) per manufacturer instructions to each well.
- Incubate for 30 min at RT in the dark.
- Add 50 µL stop solution.
- Measure absorbance at 490 nm on a multi-plate reader.

#### 3.2.10 Pro-inflammatory markers (secreted proteins)

- Store the remaining 400 µL basal medium at –80°C (in the original plate + sealing tape) until analysis.

## 4 Cell culture maintenance after exposure (tissue Set 5)

### 4.1 Day 5 (day 1 post-4 day exposure) CBF/AAA measurements

- Warm MucilAir Medium to 37°C
- 20 hours after end of the previous exposure (i.e., the same time exposure started), transfer the plate with tissue inserts from the incubator to the microscope and conduct CBF/AAA measurements.
- Use the Cilia-X software to measure CBF/AAA.
- Transfer the plate with tissue inserts to the laminar flow cabinet.
- Using sterile forceps, place tissues in a new plate with 700 µL fresh, warm basal medium and return them to the incubator.
- Store the plate with old medium for LDH assay and conduct procedure as specified for the assay.

Note: use the positive control sample from the day before

### 4.2 Day 7 (day 3 post-4 day exposure): MucilAir are washed and put in new medium

- Warm MucilAir Medium and saline solution to 37°C.
- Transfer the plate with tissue inserts to the laminar flow cabinet.
- Add 250 µL of warm saline solution (or MucilAir medium) to the apical side of each insert.
- Transfer the plate back to the incubator for 5 min at 37°C.
- Pipet solution up and down two times with 100 µL pipette.
- Gently aspirate medium by slightly tilting the plate and aspirating from the side of the insert and well(apically) and discard aspirate.
- Using sterile forceps, place tissues in a new plate with 700 µl fresh warm basal medium and return tissues to incubator.

### 4.3 Day 9 (day 5 post-4-day exposure): MucilAir are washed and put in new medium

- Warm MucilAir Medium and saline solution to 37°C.
- Transfer the plate with tissue inserts to the laminar flow cabinet.
- Add 250 µL of warm saline solution (or MucilAir medium) to the apical side of each insert.
- Transfer the plate back to the incubator for 5 min at 37°C.
- Pipet solution up and down two times with 100 µL pipette.
- Gently aspirate medium by slightly tilting the plate and aspirating from the side of the insert and well (apically) and discard aspirate.
- Using sterile forceps, place tissues in a new plate with 700 µL fresh warm basal medium and return tissues to the incubator.

## 5 Assays for recovery time point

### 5.1 Day 11 (7 days after exposure on day 4):

- Conduct preparations for dosing and CBF/AAA as specified for the assay. Only positive controls will be exposed (LDH, LPS).
- Conduct 4 hour exposure for positive controls (LDH, LPS) and proceed directly to procedures described under exposure termination and assays.
